# Endothelial Lipase Is Involved in Cold-Induced High-Density Lipoprotein Turnover and Reverse Cholesterol Transport in Mice

**DOI:** 10.3389/fcvm.2021.628235

**Published:** 2021-03-05

**Authors:** Nicola Schaltenberg, Clara John, Markus Heine, Friederike Haumann, Franz Rinninger, Ludger Scheja, Joerg Heeren, Anna Worthmann

**Affiliations:** ^1^Department of Biochemistry and Molecular Cell Biology, University Medical Center Hamburg-Eppendorf, Hamburg, Germany; ^2^Department of General Visceral and Thoracic Surgery, University Medical Center Hamburg-Eppendorf, Hamburg, Germany; ^3^Department of Internal Medicine III, University Medical Center Hamburg Eppendorf, Hamburg, Germany

**Keywords:** brown adipose tissue, HDL, endothelial lipase, cholesterol, lipidomics

## Abstract

The physiologic activation of thermogenic brown and white adipose tissues (BAT/WAT) by cold exposure triggers heat production by adaptive thermogenesis, a process known to ameliorate hyperlipidemia and protect from atherosclerosis. Mechanistically, it has been shown that thermogenic activation increases lipoprotein lipase (LPL)-dependent hydrolysis of triglyceride-rich lipoproteins (TRL) and accelerates the generation of cholesterol-enriched remnants and high-density lipoprotein (HDL), which promotes cholesterol flux from the periphery to the liver. HDL is also subjected to hydrolysis by endothelial lipase (EL) (encoded by *LIPG*). Genome-wide association studies have identified various variants of EL that are associated with altered HDL cholesterol levels. However, a potential role of EL in BAT-mediated HDL metabolism has not been investigated so far. In the present study, we show that in mice, cold-stimulated activation of thermogenic adipocytes induced expression of *Lipg* in BAT and inguinal WAT but that loss of *Lipg* did not affect gene expression of thermogenic markers. Furthermore, in both wild type (WT) and *Lipg*-deficient mice, activation of thermogenesis resulted in a decline of HDL cholesterol levels. However, cold-induced remodeling of the HDL lipid composition was different between WT and *Lipg*-deficient mice. Notably, radioactive tracer studies with double-labeled HDL indicated that cold-induced hepatic HDL cholesterol clearance was lower in *Lipg*-deficient mice. Moreover, this reduced clearance was associated with impaired macrophage-to-feces cholesterol transport. Overall, these data indicate that EL is a determinant of HDL lipid composition, cholesterol flux, and HDL turnover in conditions of high thermogenic activity.

## Introduction

Brown adipose tissue (BAT) produces heat to defend mammals against cold stress (1). In response to temperatures below thermoneutrality, adaptive thermogenesis is enabled in thermogenic brown and beige adipocytes within the BAT and white adipose tissue (WAT), respectively (2, 3). Due to their great need for energy, thermogenic adipocytes take up nutrients from the circulation and thereby impact on plasma glucose and lipid levels, which makes them an appealing therapeutic target for the treatment of metabolic disorders (4, 5). Contrary to an earlier study showing that the activation of BAT exacerbates atherosclerosis in mice lacking the low-density lipoprotein (LDL)-receptor or apolipoprotein E (ApoE) (6), we have shown that BAT activation not only corrects hypertriglyceridemia (7) but also reduces plasma cholesterol levels and atherosclerosis in transgenic mice expressing both a loss-of-function variant of human apolipoprotein E (APOE^*^3-Leiden; E3L) and the human cholesteryl ester transfer protein (E3L.CETP mice) (8). Furthermore, we showed that the activation of adaptive thermogenesis stimulates reverse cholesterol transport (RCT) and accelerates high-density lipoprotein (HDL) turnover in mice, which was accompanied by characteristic changes in the HDL lipid composition (9). While we identified lipoprotein lipase (LPL) to be crucial for this process, the impact of other lipases on HDL lipid remodeling and turnover after activation of adaptive thermogenesis remains elusive. Another lipase of the triglyceride (TG) lipase gene family that is present in vascular lumen of metabolically active organs is the endothelial lipase (EL) (encoded by *LIPG*) (10). Variants in human *LIPG* were found to be associated with altered plasma HDL cholesterol levels (11, 12) and several genome-wide association studies identified and confirmed associations of SNPs in *LIPG* to HDL cholesterol (HDL-C) levels (13–16). EL is generally expressed by endothelial cells, shows mainly phospholipase and little TG lipase activity (17) and preferentially hydrolyzes HDL lipids (18, 19). In addition, EL mediates binding and uptake of HDL and its cholesteryl esters (CE) into the liver independently of its lipolytic activity (20). While deletion or blocking of EL results in increased HDL-C levels (18, 21, 22), overexpression of EL decreases HDL-C levels (18, 23). EL has also been implicated in the modulation of cholesterol efflux capacity (24–27), HDL properties (28, 29), and atherosclerosis development (30, 31). Besides its role for HDL metabolism, EL has recently been shown to be involved in very-low-density lipoprotein (VLDL) processing and concomitant LDL-C lowering in response to angiopoietin-like 3 inhibition (32). Moreover, a high *LIPG* expression was found in breast cancer and EL-mediated lipid uptake and lipid storage in response to oxidative stress promoted breast cancer growth and tumor progression (33, 34). Of note, inhibition of EL phospholipase activity suppressed tumor formation in breast cancer (35).

As HDL metabolism is strongly affected by both, the EL and the activation of thermogenesis, we hypothesize that EL is involved in the promotion of HDL turnover and HDL lipid remodeling observed in response to activation of thermogenic adipose tissues. In the present study, we show that upon activation of thermogenesis, EL was induced in BAT and inguinal WAT (ingWAT) of wild type (WT) mice. In addition, we found that although the loss of EL did not attenuate reductions in plasma lipid and especially HDL-C levels, it impaired HDL lipid remodeling and diminished HDL turnover and macrophage-to-feces disposal of cholesterol. These results suggest that EL promotes acceleration of HDL metabolism after activation of adaptive thermogenesis and that not HDL-C levels *per se* but rather HDL characteristics and its lipid composition determine the cholesterol efflux capacity of HDL.

## Materials and Methods

### Experimental Animals, Housing Conditions, Diets, and Animal Experiments

All animal experiments were approved by the Animal Welfare Officers of University Medical Center Hamburg-Eppendorf (UKE) and Behörde für Gesundheit und Verbraucherschutz Hamburg. Mice lacking EL globally (*Lipg*^−/−^) were purchased from The Jackson Laboratory, backcrossed to C57BL/6J WT for at least seven generations, and mice homozygous for *Lipg* deletion were used for breeding. WT littermates served as controls. Both C57BL/6J WT control- and *Lipg*^−/−^ mice were housed at the animal facility of the UKE at 22°C with a day–night cycle of 12 h with *ad libitum* access to food and water. The mice were fed a regular chow diet (P1324, Altromin, Germany) or a western-type diet (WTD) (Sniff EF R/M acc.TD88137 mod). For the experiments, 8- to 12-week-old male mice were housed for 7 days in a thermoneutral (30°C) or cold (6°C) environment. Tissue and blood collections were performed after a 4-h fasting period. The mice were anesthetized with a lethal dose (15 μl/g mouse bodyweight) of a mixture containing ketamin (25 mg/ml)/xylazin (0.2%) in 0.9% NaCl. Blood was withdrawn by cardiac puncture with syringes containing 5 μl of 0.5 M EDTA for plasma preparation. Subsequently, the animals were perfused with 5 ml of ice-cold PBS containing 10 U/ml heparin. The organs were harvested and immediately conserved either in TriFast™TRIfast® reagent (Peqlab) for RNA analysis or snap-frozen in liquid nitrogen and stored at −80°C for further processing.

### Gene Expression Analysis

Tissue samples were homogenized in TriFast™ using a Qiagen Tissue Lyzer followed by extraction of nucleic acids by chloroform before RNA was purified using RNA Purification Kit NucleoSpin® RNA II (MACHEREY-NAGEL) following the manufacturer's instructions. Complementary DNA was synthesized by means of SuperScript® III Reverse Transcriptase (Invitrogen). Quantitative real-time PCR for indicated genes were run on a 7900HT sequence detection system (Applied Biosystems) using TaqManAssay-on-Demand primer sets (Applied Biosystems, *Abca1*: Mm00442646_m1, *Abcg1*: Mm00437390_m1, *Abcg5*: Mm00446249_m1, Abcg8: Mm00445970_m1, *Cd36*: Mm00432403_m1, *Cyp7a1*: Mm00484150_m1, *Cyp7b*1: Mm00484157_m1, *Dio2*: Mm00515664_m1, *Elovl3*: Mm00468164_m1, *Slc2a4*: Mm01245502_m1, *Hmgcr*: Mm01282499_m1, *Ldlr*: Mm00440169_m1, *Lpl*: Mm00434764_m1, *Lrp1*: Mm00464608_m1, *Ppargc1a*: Mm00447183_m1, *Scarb1*: Mm00450234_m1, *Ucp1*: Mm00494069_m1). Cycle thresholds (Cts) were normalized to TATA-box-binding protein (Tbp) housekeeper levels by using the ΔΔCt method.

### Plasma Analysis

EDTA-spiked blood samples were centrifuged for 10 min at 10,000 rpm and 4°C to yield plasma. To assess plasma cholesterol and triglycerides commercial kits (Roche) were adapted to 96-well microtiter plates. Precipath® was used as a standard for cholesterol as well as triglycerides. Lipoprotein profiling was conducted by fast-performance liquid chromatography (FPLC). Pooled plasma (200 μl) was separated on a Superose™ 6 Increase 10/300 GL column (GE Healthcare) with a flow rate of 0.4 ml/min. Forty fractions (volume of faction 0.5 ml) were collected. Triglycerides as well as cholesterol concentrations were measured in each fraction. To yield HDL, 0.35 ml from fractions 20–22 was pooled.

### Lipidomic Analysis

For HDL lipidomic analysis, individual FPLC runs were performed for every mouse and FPLC fractions 20–22 of each mouse were pooled as HDL. Briefly, lipids from HDL were extracted by the method of Bligh and Dyer (36). One milliliter methanol, 0.05 ml of 1:10 diluted internal standard mix (Table 1) and 0.7 ml of chloroform were added to 0.32 ml of the HDL solution and mixed by vortexing for 30 s. After addition of 1.1 ml chloroform and 0.9 ml water, samples were again mixed for 30 s by vortexing. After centrifugation at 3,000 g and 4°C for 15 min, 1.6 ml of the lower organic phase was transferred into a new glass vial, and the solvent was evaporated to dryness by vacuum centrifugation. Dry lipid extracts were resuspended in 0.08 ml of eluent B and transferred into glass vials. Lipidomic analyses were conducted on a Dionex3000 UPLC [Column: Kinetex C18, 150 × 2.1 mm; 1.7 μm (Phenomenex)] coupled to an ESI-UHR-Q-TOF mass spectrometer (maXis3G, Bruker Daltonik). Data was acquired in high-resolution, full scan MS mode using collisional induced dissociation fragmentation. Chromatography was performed at a flow rate of 0.3 ml min^−1^ using the following gradient: Starting from 80%, eluent B was increased in 2 min to 87%, held constant for 6 min, again increased in 2 min to 95%, followed by another increase to 99% in 10 min. After 1 min of 99% (isocratic), eluent B was reduced to 80% in 1 min and kept constant for another 3 min. Eluent A consisted of ultra-pure water enriched with 5 mM NH_4_Ac, eluent B of MeOH/IPA, 4/6 (vol/vol) enriched with 5 mM NH_4_Ac. Column temperature was maintained at 55°C. The mass spectrometer was operated in positive ionization mode: A capillary voltage of 4.5 kV and an end plate offset voltage of −500 V were applied. The inlet LC flow was nebulized using nitrogen gas (2 bar), the dry temperature was kept at 190°C. Data were acquired over a mass range of 100–1,000 Da for both MS and MS/MS modes. Nitrogen was used for collisional induced dissociation with collision energies between 14 and 35. Obtained spectra were externally calibrated using ESI-L tuning mix (Agilent Technologies) running via syringe pump at 20 μl h^−1^. Further internal calibration was performed for each sample by using the lockmass hexakis (1H, 1H, 2H-perfluoroethoxy) phosphazene (Apollo Scientific Limited). UPLC-MS data were processed using the manufacturer software (DataAnalysis 4.0 and TargetAnalysis 1.3). Peak areas of individual lipid species were calculated by comparing the individual peak areas with those of corresponding internal standards for determining the final concentrations. Only peak areas that were within the range of external calibration curves were considered for quantification. Lipid species were identified by means of standard substances, MS/MS-spectra, and the LIPID MAPS database (https://www.lipidmaps.org/tools/ms/). Upon reasonable request, lipidomic raw data will be made available.

**Table 1:** Components of internal standard for lipidomic analysis.

**Internal standard components (in chloroform/methanol, 2/1, v/v)**
- 1,2-dipentadecanoyl-sn-glycero-3-phosphatidylcholine (118.85 μg/ml)
- cholest-5-en-3ß-yl heptadecanoate (593.27 μg/ml)
- Glyceryltritridecanoate (118.85 μg/ml)
- N-heptadecanoyl-D-erythro-sphingosine (118.85 μg/ml)
- 1-heptadecanoyl-2-hydroxy-sn-glycero-3-phosphocholine (118.85 μg/ml)
- 1,2-diheptadecanoyl-sn-glycero-3-phosphoethanolamine (118.85 μg/ml)
- 1-o-pentadecanyl-3-(9Z-octadecenoyl)
- sn-glycerol (118.85 μg/ml)
- 1,3-diheptadecanoyl glycerol (118.85 μg/ml)
- 1,2-di-O-tridecyl-sn-glycero-3-phosphocholine (118.85 μg/ml)
- N-heptadecanoyl-D-erythro-sphingosylphosphorylcholine (118.85 μg/ml)
- 1,2-ditetradecanoyl-sn-glycero-3-phospho-L-serine, sodium salt (118.85 μg/ml)
- 1,2-diheptadecanoyl-*sn*-glycero-3-phosphate, sodium salt (118.85 μg/ml)
- heptadecanoic acid (237.31 μg/ml)

### High-Density Lipoprotein Turnover

HDL was isolated by sequential ultracentrifugation (d = 1.063–1.21 g/ml) as described by Havel (37) from C57BL/6J WT mice, which were fasted 4 h before blood withdrawal. HDL was then double-labeled with ^125^I-tyramine cellobiose (125I-TC) in the apolipoprotein moiety and with ^3^H-cholesteryl oleoyl ether (CEt) in the lipoprotein core as described before (38). Briefly, human plasma CETP was used to introduce ^3^H-CEt into ^125^I-TC-HDL by exchange from donor liposomal particles, containing ^3^H-CEt. The final ^125^I-TC-/^3^H-CEt-HDL particles were dialyzed against PBS (pH 7.4, 4°C) with added 1 mM EDTA. In the following, ^25^I-TC-/^3^H-CEt-HDL (30 mg HDL protein per mouse; ca. 39 kBq 125I-TC and 33 kBq 3H-CEt, respectively) were injected into the tail vein of 4-h fasted mice for analysis of plasma decay and organ uptake of radiolabeled HDL. Blood samples were collected at given times after injection: 10 and 30 min; 1, 2, and 5 h. While plasma aliquots and tissues were directly assayed for ^125^I radioactivity, ^3^H-radioactivity was analyzed by scintillation counting after lipid extraction using the method of Dole (39).

### Reverse Cholesterol Transport Assay

RCT assay was performed according to the method of Rader et al. (40). Four days before isolation of peritoneal macrophages, *Ldlr*^–/–^ mice were injected with 2 ml thioglycollate into the peritoneal cavity. Macrophages were then isolated by peritoneal lavage with warm DMEM and plated. After 4 h, macrophages were radiolabeled *ex vivo* with tracer ^3^H-cholesterol (100 kBq corresponding to 1.5 × 10^6^ cells per mouse) followed by overnight incubation with acetylated LDL (20 μg/ml) to load them with cholesterol. Needles (Braun), 0.55 25 mm 24 G × 1^n^ Gr. 17, were used for the intraperitoneal injection of 1 × 10^6^ radiolabeled macrophages to reduce shear stress. After injection, the mice were kept in special cages for the entire duration of the experiment to prevent them from tampering with the feces. Feces, tissue, and blood collections were performed 48 h after macrophage injection. ^3^H-radioactivity of plasma, feces, and organs were measured as described above.

### Statistical Analysis

All statistics were performed using GraphPad Prism7 (StatCon). When analyzing small group sizes (*n* = 3), the data were logarithmically transformed before statistical analysis. All groups were compared to all other groups. When comparing two groups, significance was calculated using non-paired two-tailed Student's *t*-test. When comparing more than two groups, two-way ANOVA followed by *post hoc* testing (Tukey correction) was conducted if not stated otherwise. All values in the figure panels show mean values ± SEM. *P*-values lower than 0.05 were considered statistically significant. When comparing two groups, statistical significance was displayed as *P*-values. For clarity, when comparing four groups, statistical significance is indicated by the connecting letters report, where shared letters indicate groups that are not significantly different from each other, while different letters indicate a statistical difference.

## Results

### Endothelial Lipase Induced by Cold Does Not Affect Thermogenic Gene Expression

**Figure 1:**
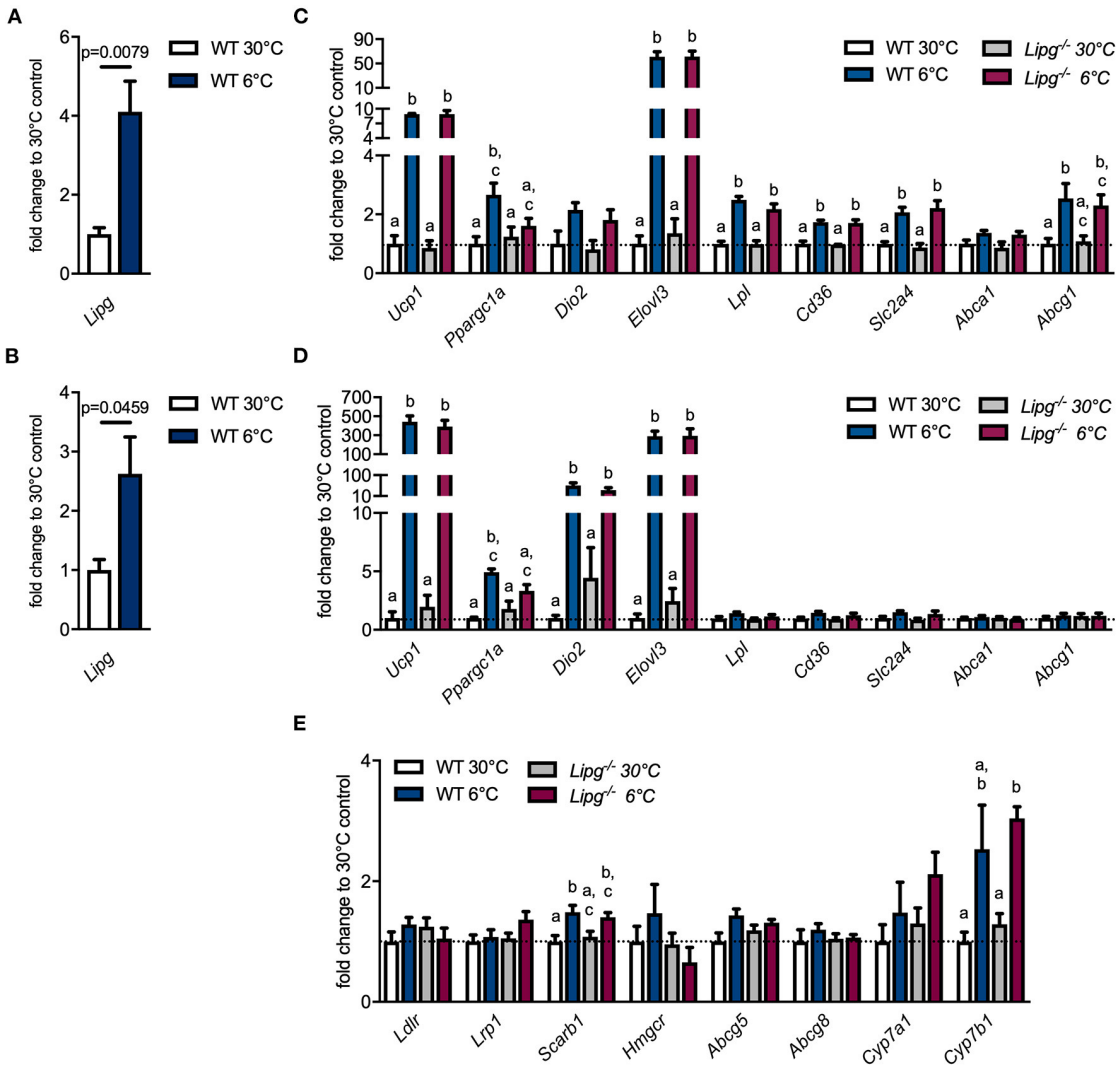
Loss of endothelial lipase (EL) does not compromise thermogenic response. Wild type (WT) mice were housed for 7 days either in a thermoneutral (30°C) or cold (6°C) environment and gene expression of *Lipg* was measured in iBAT **(A)** and ingWAT **(B)**. WT and *Lipg*^−/−^ mice were housed for 7 days either in a thermoneutral (30°C) or cold (6°C) environment and gene expression of indicated genes was measured in iBAT **(C)**, ingWAT **(D)** and liver **(E)**. iBAT, interscapular brown adipose tissue (BAT); ingWAT, inguinal white adipose tissue (WAT). Each bar represents the mean of four to six mice per group ± SEM. Statistics were performed using two-way ANOVA and *p*-values lower than 0.05 were considered statistically significant; different letters indicate significant differences between groups.

During cold stress, thermogenic adipocytes within BAT and WAT rely on excessive nutrient uptake in order to maintain heat production. LPL is upregulated and ensures the release of fatty acids from triglyceride-rich lipoprotein (TRL) particles to deliver sufficient fuel. Furthermore, in states of adaptive thermogenesis, we have identified LPL as a central player in driving cholesterol excretion and promoting HDL metabolism (9). Another enzyme involved in remodeling and metabolism of HDL particles is EL. However, its regulation and function during adaptive thermogenesis remains elusive. In order to investigate the impact of cold intervention on EL, we housed C56Bl/6J mice at thermoneutrality (30°C) or in a cold environment (6°C) for 7 days and found profound upregulation of *Lipg* expression in interscapular BAT (iBAT) and ingWAT of cold-housed mice (Figures 1A,B). Next, we studied if loss of EL affects thermogenesis in BAT and WAT of 1-week thermoneutral- (30°C) or cold-housed (6°C) WT and *Lipg*^−/−^ mice. In iBAT, cold housing resulted in higher expression of thermogenic marker genes such as *uncoupling protein 1* (*Ucp1), peroxisome proliferator-activated receptor gamma (Ppargc1a), iodothyronine deiodinase 2* (*Dio2)*, and *fatty acid elongase3* (*Elovl3*) irrespective of the genotype. Only *Ppargc1a* did not exhibit a significant induction in cold-housed *Lipg*^−/−^ mice (Figure 1C). In the same line, genes important for processing and uptake of lipids [*lipoprotein lipase* (*Lpl*)*, cluster of differentiation 36* (*Cd36*)] and glucose [*solute carrier family 2 member 4* (*Slc2a4*)] were elevated in WT and *Lipg*^−/−^ mice in response to cold (Figure 1C). Additionally, expression of genes encoding cholesterol excretion transporters was slightly higher for *ATP binding cassette subfamily A member 1* (*Abca1*) and significantly higher for *ATP binding cassette subfamily G member 1* (*Abcg1*), after cold housing (Figure 1C). Similar to observed alterations in iBAT, cold housing also increased thermogenic gene expression in ingWAT. In WT- and *Lipg*^−/−^ mice, *Ucp1, Ppargc1a, Dio2*, and *Elovl3* were profoundly upregulated while genes mediating nutrient import and export (*Lpl, Cd36, Slc2a4, Abca1, Abcg1*) remained mainly unaffected (Figure 1D). Since activation of BAT by cold not only stimulates lipid metabolism in adipose tissues but also affects systemic lipoprotein trafficking and enhances RCT (7, 9), we additionally analyzed hepatic gene expression given the pivotal role of the liver in lipoprotein metabolism. Whereas, gene expression of *LDL receptor* (*Ldlr*) and *LDL receptor-related protein 1* (*Lrp1*), mediating uptake of LDL and remnant particles, was unaltered by genotype or housing conditions, expression of *scavenger receptor class B member 1* (*Scarb1*) facilitating HDL-C uptake was elevated in cold-housed WT and *Lipg*^−/−^ mice (Figure 1E). Additionally, expression of *3-hydroxy-3-methylglutaryl-Coenzyme A reductase (Hmgcr), ATP binding cassette subfamily G member 5* (*Abgc5*), and *ATP binding cassette subfamily G member 8* (*Abcg8)*, mediators of cholesterol synthesis and its excretion into bile, was not significantly affected neither by cold treatment nor by loss of EL (Figure 1E). While expression of the classical BA synthesis gene cholesterol 7 alpha-hydroxylase (encoded by *Cyp7a1*) was only slightly increased in response to cold housing, expression of the gene mediating alternative BA synthesis, cytochrome P450 family 7 subfamily B member 1 (encoded by *Cyp7b1*) was higher after cold housing (Figure 1E). This is in line with previous studies (41). Both BA syntheses genes were not affected by *Lipg* loss. In summary, although *Lipg* was highly induced in thermogenic fat after cold intervention, its loss did not affect expression of genes involved in thermogenesis and lipoprotein metabolism.

### Loss of Endothelial Lipase Results in Accumulation of High-Density Lipoprotein Lipids and Attenuates Cold-Induced High-Density Lipoprotein Remodeling

**Figure 2:**
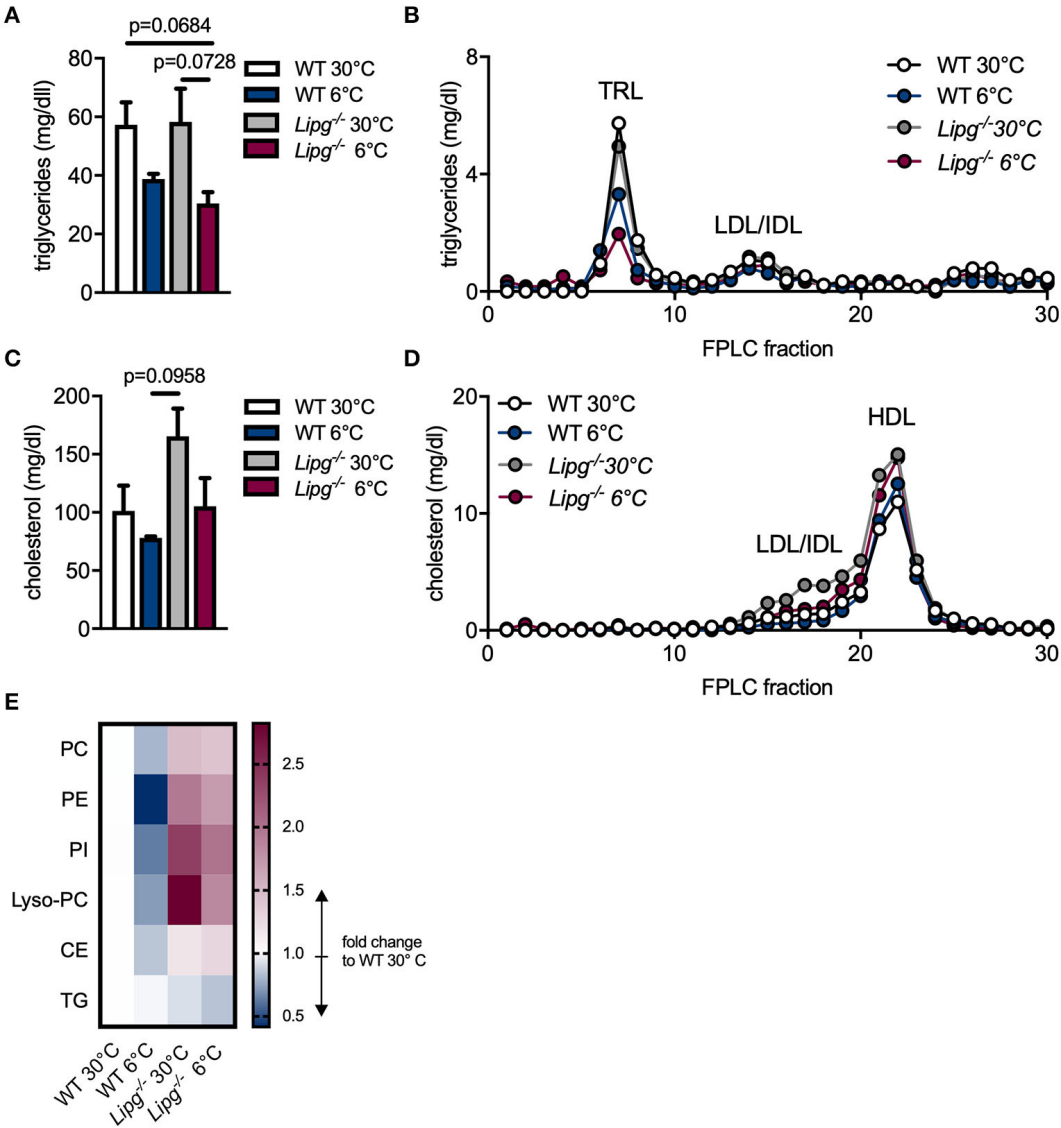
Loss of EL increases high-density lipoprotein (HDL) cholesterol and impairs HDL lipid remodeling. WT and *Lipg*^−/−^ mice were housed for 7 days either in a thermoneutral (30°C) or cold (6°C) environment and plasma samples were taken after a 4 h fasting period. Triglyceride **(A,B)** and cholesterol **(C,D)** levels were measured in total plasma **(A,C)** and fast-performance liquid chromatography (FPLC) fractions **(B,D)**; TRL, triglyceride-rich lipoprotein; LDL, low density lipoprotein; IDL, intermediate-size lipoprotein; HDL, high density lipoprotein. For HDL lipidomic analysis, individual FPLCs were run for each mouse (*n* = 4/group) and per mouse FPLC fractions 20–22 were pooled as HDL. Alterations in HDL lipid levels are shown for indicated lipid classes in comparison to WT 30°C, PC, phosphatidyl-cholines; PE, phosphatidyl- ethanolamines; PI, phosphatidyl-inositols; Lyso-PC, lysophosphatidyl-cholines; CE, cholesterol ester; TG, triglycerides. **(E)**. Each bar/dot represents the mean of three mice per group ± SEM **(A–D)**. Statistics were performed using two-way ANOVA and *p*-values lower than 0.05 were considered statistically significant. Data from **(A,B)** were subjected to logarithmic transformation before two-way ANOVA.

During cold adaption, lipoprotein metabolism is highly accelerated to provide fuel for BATs. Not only does activated BAT assimilate tremendous amounts of TG for heat production and thus substantially lowers plasma TG levels, it dramatically impacts on cholesterol and especially HDL metabolism (7–9). Since EL is induced in BAT of cold-housed mice and is known to favor HDL lipids as substrates, we speculated that EL contributes to the observed effects of BAT on lipoprotein and especially HDL metabolism. For this reason, we housed *Lipg*^−/−^ and control mice for 1 week either in a thermoneutral (30°C) or a cold environment, collected blood samples, measured plasma lipids, and subjected plasma to FPLC for lipoprotein analysis. TG levels were unaltered in total plasma (Figure 2A) and the TRL-fraction (Figure 2B) of thermoneutral-housed *Lipg*^−/−^ compared to thermoneutral-housed WT mice. As expected, although only by trend, cold housing resulted in lower TG levels in total plasma and TRLs of both, WT and also EL-deficient mice. In line with previous reports (18, 22), *Lipg*^−/−^ mice trended toward higher plasma cholesterol levels compared with WT controls (Figure 2C), owing to increases in HDL-C (Figure 2D). Of note, elevated cholesterol was also found in particles of intermediate size (fraction 15–18) in *Lipg*^−/−^ mice (Figure 2D). Although cold housing lowered plasma cholesterol levels in WT and *Lipg*^−/−^ mice by trend, cholesterol levels remained higher in cold-housed *Lipg*^−/−^ mice compared with their respective controls (Figure 2C). Of note, the cold-induced cholesterol reduction was mainly due to reductions in intermediate-sized lipoproteins (Figure 2D).

Upon activation of BAT, HDL lipidome is subjected to remodeling in mice and humans (9). We hypothesized, that loss of EL would impair cold-induced alterations in the HDL lipidome and thus performed LC-MS/MS-based lipidomic analysis of HDL particles isolated from WT and *Lipg*^−/−^-deficient mice housed either in a thermoneutral (30°C) or cold (6°C) environment. Irrespective of genotype, HDL-TG levels remained mostly unaffected by cold treatment (Figure 2E). In HDL particles isolated from WT mice, cold housing resulted in lower amounts of phosphatidylcholines (PC), phosphatidylethanolamines (PE), phosphatidylinositols (PI), Lyso-PC, and CEs indicating lipid remodeling (Figure 2E). In contrast, as mirrored in elevated HDL levels, all other determined HDL lipid classes except for TG, were increased in *Lipg*^−/−^ mice compared to WT mice irrespective of housing temperature (Figure 2E). Nevertheless, also in mice deficient for EL, cold housing especially reduced PI and Lyso-PC whereas PC, PE, and CE remained mainly unaffected (Figure 2E). In sum, while cold-induced reductions in cholesterol mainly occur due to reduction of intermediate-sized particles, reductions in HDL-C upon cold-housing are abrogated in *Lipg*^−/−^ mice. In the same line, cold-induced HDL lipidome remodeling is diminished in mice lacking EL.

### Cold-Induced Acceleration of High-Density Lipoprotein Turnover Is Blunted in *Lipg^−/−^* Mice

**Figure 3:**
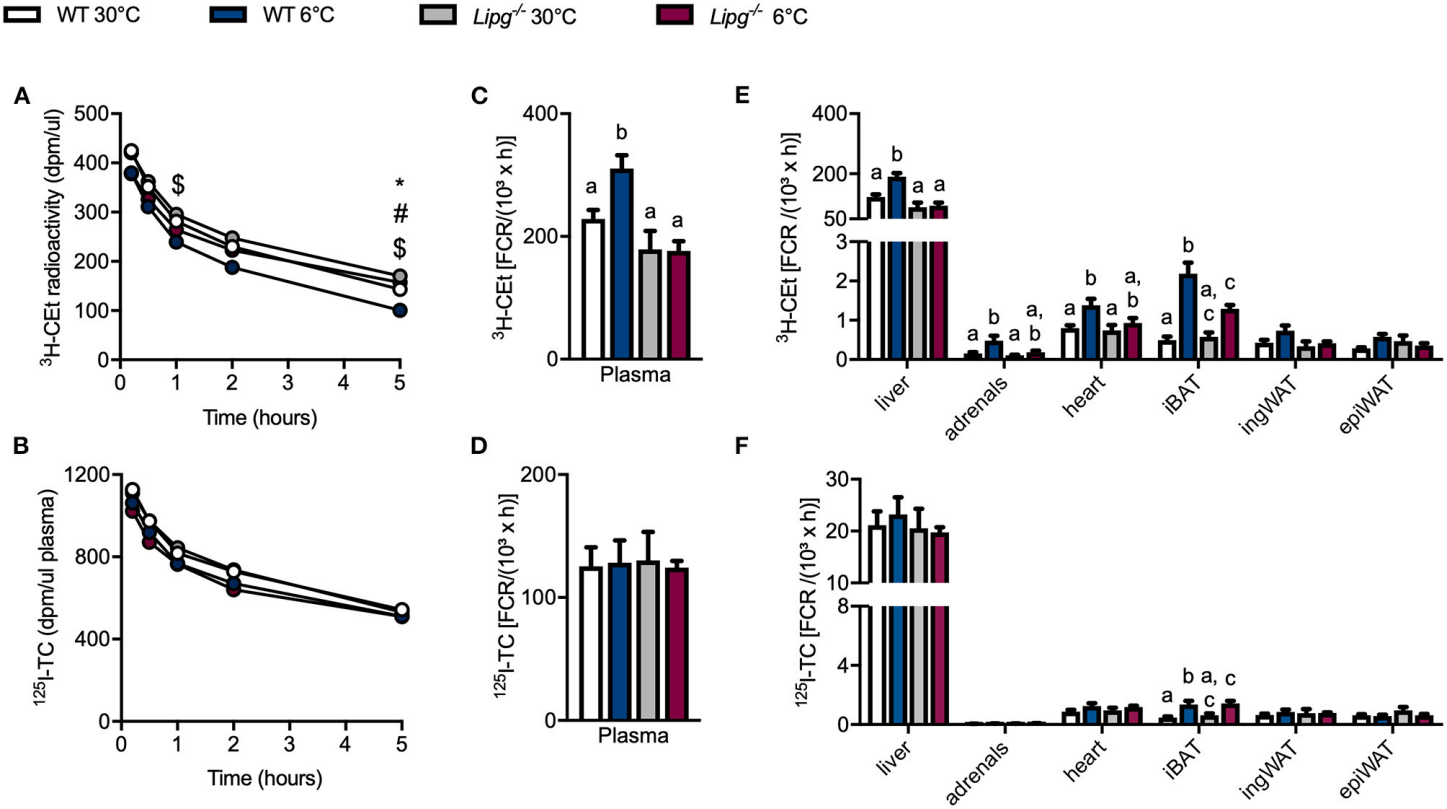
Loss of EL dampens cold-induced HDL turnover. WT and *Lipg*^−/−^ mice were housed for 7 days either in a thermoneutral (30°C) or cold (6°C) environment before they received an injection of ^125^I-tyramine cellobiose (TC) and ^3^H-cholesteryl oleoyl ether (CEt) radiolabeled HDL **(A)**. At indicated time points ^3^H-CEt activity in plasma was analyzed **(B)**. At indicated time points ^125^I-TC activity in plasma was analyzed. Five hours after injection of radiolabeled HDL fractional catabolic rate (FCR) was determined in plasma for ^3^H-CEt **(C)** and ^125^I-TC **(D)** and in organs for ^3^H-CEt **(E)** and ^125^I-TC **(F)**. iBAT, interscapular BAT; ingWAT, inguinal WAT; epiWAT, epididymal WAT. Each bar represents the mean of five to six mice per group ± SEM. Statistics were performed using two-way ANOVA and *p*-values lower than 0.05 were considered statistically significant and are indicated as follows for **(A,B)**: *WT30 vs. KO 30; #WT6 vs. KO 6; $WT30 vs. WT 6; and for **(C–F)**: different letters indicate significant differences between groups.

To study the consequences of impaired HDL remodeling in *Lipg*^−/−^ mice, we performed HDL turnover studies. For this purpose, WT and *Lipg*^−/−^ mice were housed at thermoneutral (30°C) or cold (6°C) conditions for 1 week, and subsequently intravenously injected with radioactively labeled HDL particles. Here, protein moiety of the particles was labeled with ^125^I tyramine cellobiose (^125^I-TC) and the lipid core was labeled with ^3^H-cholesteryl ether (^3^H-CEt). Clearance of HDL from the circulation was monitored by blood sampling at indicated time points and uptake of radioactive tracers into organs was measured 5 h after injection. In both, WT and *Lipg*^−/−^ mice, radioactive tracers were cleared over experimental duration. While WT mice exhibited accelerated plasma clearance of ^3^H-CEt upon cold treatment, this effect was absent in *Lipg*^−/−^ mice (Figure 3A). Plasma clearance of ^125^I-TC was neither modulated by genotype nor housing condition (Figure 3B). Plasma fractional catabolic rate (FCR) calculated from decay curves showed the same picture: in WT mice, cold housing increased the plasma FCR for HDL-associated ^3^H-CEt (Figure 3C) while ^125^I-TC remained the same (Figure 3D). Contrary, in *Lipg*^−/−^ mice, plasma ^3^H-CEt (Figure 3C) and ^125^I-TC (Figure 3D) FCR remained unchanged upon cold exposure. Furthermore, we determined the fraction of HDL particles cleared from the circulation into organs, the so-called organ FCR. We found, that cold housing increased liver FCR of ^3^H-CEt (Figure 3E) but not of ^125^I-TC (Figure 3F) in WT but not in *Lipg*^−/−^ mice. Additionally, cold housing also increased ^3^H-CEt FCR of adrenals and heart in WT but not EL-deficient mice, whereas ^125^I-TC FCR was unaltered (Figures 3E,F). Interestingly, in both WT and *Lipg*^−/−^ mice, activation of BAT by cold resulted in higher iBAT FCR for ^3^H-CEt (Figure 3B) and ^125^I-TC (Figure 3C), suggesting enhanced uptake of whole HDL particles into BAT. No changes in ^125^I-TC and ^3^H-CEt FCR were observed for ingWAT and epididymal WAT (epiWAT). Thus, in WT mice, cold exposure resulted in accelerated clearance of HDL-derived CEs from the circulation and increased uptake of CEs into the iBAT and the liver, while especially the liver uptake was blunted in the in *Lipg*^−/−^ mice.

### Ablation of Endothelial Lipase Diminishes Cold-Stimulated Macrophage-to-Feces Cholesterol Transport

**Figure 4:**
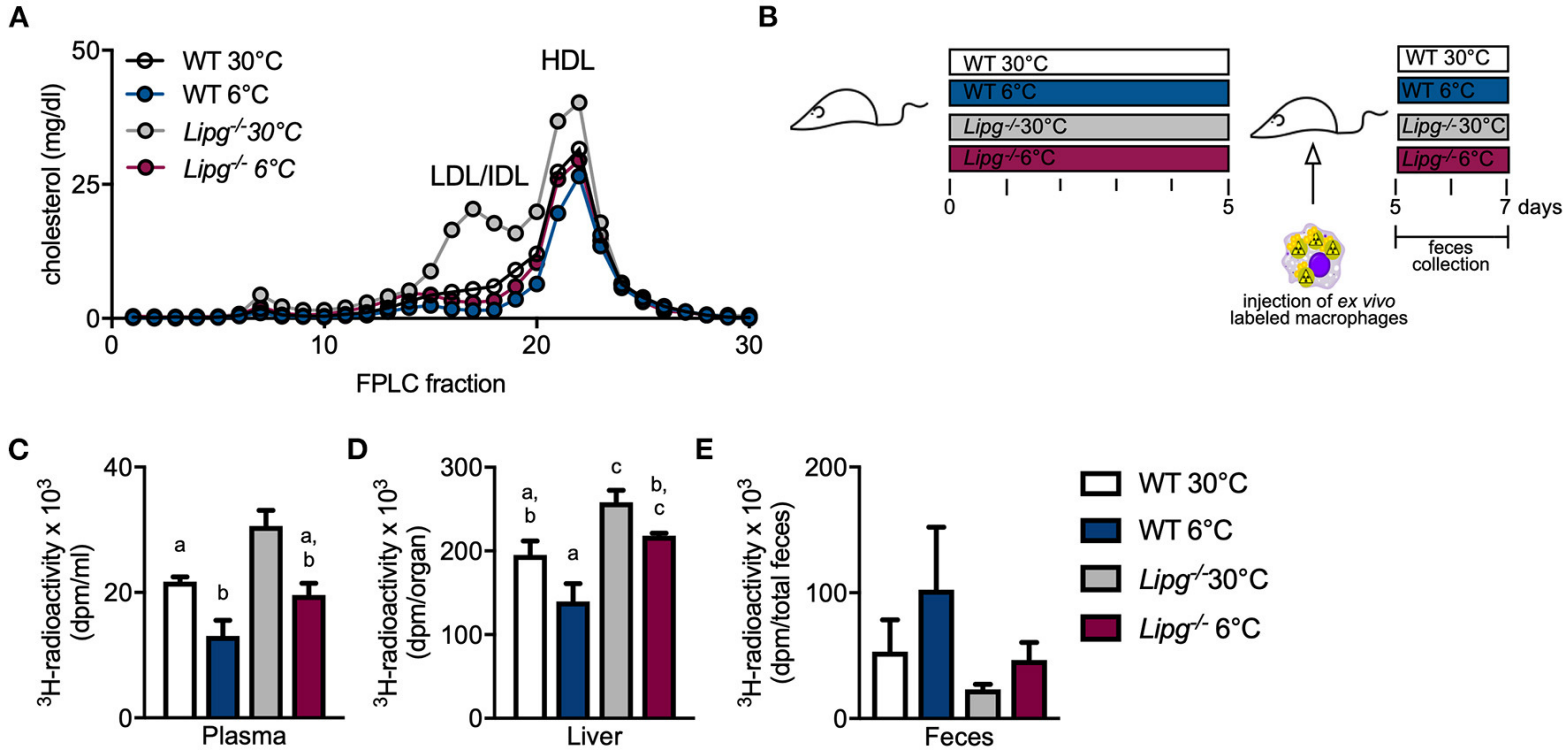
EL-deficient mice present diminished reverse cholesterol transport (RCT) independent of housing temperature. Plasma FPLC profile of western-type diet-fed WT and *Lipg*^−/−^ mice after 7 days of housing at 6 or 30°C **(A)**. Five days prior to injection of *ex vivo* ^3^H-radiolabeled macrophages, WT and *Lipg*^−/−^ mice were housed in a thermoneutral (30°C) or cold (6°C) environment. Then, the mice remained on indicated housing temperature and macrophages were allowed 48 h of circulation before mice were sacrificed **(B)**. Clearance from plasma is determined by ^3^H-cholesterol radioactivity in plasma **(C)** and liver uptake by measuring ^3^H-cholesterol radioactivity in liver **(D)**. Excretion of ^3^H-cholesterol was measured by collecting feces during 48 h of macrophage circulation and determining ^3^H-cholesterol activity **(E)**. Each bar represents mean of five to seven mice per group ± SEM. Statistics were performed using two-way ANOVA, and *p*-values lower than 0.05 were considered statistically significant (different letters indicate significant differences between groups).

Next, we wanted to study if the decelerated HDL metabolism observed in *Lipg*^−/−^ mice also translated into impaired cholesterol excretion. For this purpose, we performed an RCT assay as described by Rader and colleagues (40). Briefly, we isolated and cultured peritoneal macrophages from *Ldlr*^–/–^ mice, loaded them *ex vivo* with LDL and ^3^H-cholesterol, and injected them intraperitoneally into WT and *Lipg*^−/−^ mice, which were housed in either thermoneutral (30°C) or cold (6°C) conditions and fed a cholesterol-enriched western-type diet (WTD) to stimulate cholesterol metabolism. Compared with chow feeding (Figure 2D), WTD feeding substantially raised plasma cholesterol levels irrespective of genotype and housing temperature (Figure 4A). Interestingly, while in all groups, the main cholesterol was found in the HDL (fractions 20–22), especially in *Lipg*^−/−^ mice housed at thermoneutrality, a big proportion of the cholesterol was found in intermediate-sized lipoproteins as observed before (Figure 4A). Mice were sacrificed 48 h after macrophage injection, and plasma, liver, and feces samples were harvested (Figure 4B). In accordance with earlier studies, we found that cold housing resulted in a marked reduction of ^3^H-cholesterol levels in the plasma (Figure 4C). Similarly, in WT mice, liver levels of macrophage-derived ^3^H-cholesterol decreased by trend (Figure 4D), whereas excretion into feces after 48 h seemed to be enhanced but failed to reach statistical significance (Figure 4E). In principle, the cold-induced alterations of ^3^H-cholesterol in plasma, liver, and feces were also observed in mice lacking EL, although less pronounced. In particular, compared with WT mice, cold-housed *Lipg*^−/−^ mice exhibited higher amounts of ^3^H-cholesterol in the plasma (Figure 4C) and the liver (Figure 4D) and fecal ^3^H-cholesterol-levels were lower (Figure 4E). In conclusion, the cold-induced acceleration of macrophage-to-feces cholesterol excretion is likely present in *Lipg*^−/−^ mice but decelerated compared with WT control mice.

## Discussion

The presence of active thermogenic fat in humans (42–46) and its impact on energy expenditure and lipid and glucose metabolism (5, 7, 8) have made it an appealing target for the treatment of obesity and associated disorders such as type 2 diabetes, dyslipidemia, and atherosclerosis. Atherosclerosis development is not only associated with high LDL- and remnant cholesterol levels but also plasma levels of HDL-C are a negative risk factor (47). In previous studies, we were able to show, that activation of brown and beige adipose tissue protects from the development of atherosclerosis by promoting the clearance of cholesterol remnant particles (8) and by increasing HDL flux and HDL-mediated cholesterol excretion (9). Furthermore, we showed that, once activated, thermogenic adipocytes provide a highly lipolytic environment in the vascular lumen resulting in lipid remodeling of HDL particles (9). Besides LPL, which we identified as pivotal player in this process (9), we speculated that also other lipases might be involved. In this study, we identified EL to be induced in thermogenic fat of cold-housed mice. We further demonstrated that in response to cold, loss of EL diminished HDL lipid remodeling and decelerated HDL turnover and HDL-mediated RCT.

Similarly as shown for LPL (7), activation of thermogenic adipose tissues by cold resulted in enhanced expression of *Lipg*. EL interacts with heparin sulfate proteoglycans and with circulating lipoproteins, thereby facilitating their clearance from the circulation (48, 49). In addition, EL is also able to directly promote lipid uptake and lipid storage, a process recently observed in breast cancer (33, 34). Hence, the here-described induction of EL during cold might also stimulate uptake of TRL particles into activated thermogenic adipocytes and promote fuel for thermogenesis. In agreement with loss of *LPL* (50), also EL deletion did not affect thermogenic gene expression in iBAT nor ingWAT. In summary, our data suggest, that EL-mediated lipid hydrolysis seems to be insignificant for proper function of thermogenic adipocytes.

In agreement with published data, the loss of EL increases plasma HDL concentrations irrespective of housing conditions (12, 18, 22). Interestingly, we found, that *Lipg*^−/−^ mice also showed higher cholesterol levels in lipoproteins of intermediate size, an effect that was even aggravated by WTD feeding. Only recently, Adam et al. made a similar observation: After inhibition of Angptl3, mice lacking both, the *Ldlr* and *Lipg*, showed an accumulation of cholesterol in the LDL fraction when compared to mice lacking *Ldlr* only. These data indicate that EL not only affects HDL-C but also LDL-C/IDL-C (as referred to by Adam et al. (32) or us respectively). Nevertheless, activation of thermogenic fat reduced cholesterol levels (both IDL-C/LDL-C and HDL-C) in *Lipg*^−/−^ mice, although not as strong as in WT mice. As EL-mediated PL hydrolysis has been shown to remodel HDL lipids and reduce HDL particle size (22, 51), we speculated that cold-induced remodeling of the HDL lipidome (9) might also partly depend on EL. The here-presented results support this hypothesis. In contrast to control mice, irrespective of housing temperature, mice lacking EL had higher levels of PC, PE, and PI in the HDL fraction. This is in line with a study of Schilcher et al. reporting decreased levels of PC, PE, and PI in HDL particles upon EL overexpression (29). Importantly, while in WT mice, cold housing resulted in dramatic reductions in PC, PE, PI, and Lyso-PC, these alterations were diminished in HDL particles of *Lipg*^−/−^ mice. EL-mediated HDL remodeling and reductions in HDL particle size have been shown to trigger clearance of HDL particles from the circulation (51–53). Furthermore, EL particularly promotes hepatic clearance of HDL-C via SR-B1 (52, 53). In line with the reduced remodeling of HDL particles in both warm- and cold-housed *Lipg*^−/−^ mice compared with their WT controls, we detected lower selective clearance and uptake of cholesterol into the liver, and an accumulation of cholesterol in the plasma. Especially the enhanced cholesterol clearance, which was observed in WT mice in response to activation of thermogenic fat, was absent in *Lipg*^−/−^ mice. Thus, the results from the HDL turnover studies suggest that upon activation of thermogenic fat, EL contributes to accelerated metabolic flux of HDL-C and subsequent hepatic clearance of cholesterol. Consequently, we also detected lower excretion of macrophage-derived cholesterol into the feces of mice lacking EL. However, as RCT was still higher in cold-housed *Lipg*^−/−^ mice compared to their thermoneutral-housed counterparts, EL is most likely involved but not crucial during accelerated cholesterol removal in response to the activation of thermogenic fat. These findings add another piece to the contradictory puzzle on the role of EL for RCT and HDL cholesterol efflux capacity (25, 27, 29, 54).

Of note, in agreement with the finding that high levels of HDL-C are not atheroprotective *per se* (55, 56) and may even increase the risk for CVD (57), the findings presented here emphasize the revised HDL cholesterol hypothesis (58) and show that HDL quantity does not equal HDL functionality. A reasonable measure of HDL function is not HDL quantity but rather HDL cholesterol efflux capacity. The here-presented results are in line and suggest that although loss of *Lipg* results in increased HDL levels even after activation of thermogenic fat, these higher HDL levels do not translate into enhanced HDL function. Altogether, cold-induced HDL lipid remodeling, accelerated HDL metabolism, and cholesterol excretion is diminished by EL deficiency.

## Data Availability Statement

The raw data supporting the conclusions of this article will be made available by the authors, without undue reservation.

## Ethics Statement

The animal study was reviewed and approved by Behörde für Gesundheit und Verbraucherschutz Hamburg Billstraße 80 20539 Hamburg.

## Author Contributions

NS, CJ, JH, and AW planned the project and were involved in all aspects of the experiments. NS and AW wrote the manuscript. NS, CJ, MH, and FH performed mouse experiments. FR was involved in the HDL-turnover studies. LS helped design the study. All authors discussed the results and commented on the manuscript.

## Conflict of Interest

The authors declare that the research was conducted in the absence of any commercial or financial relationships that could be construed as a potential conflict of interest.
